# Supplementary material for: CD19xCD3 T cell engager blinatumomab effective in refractory generalized myasthenic syndromes
Source: Mol Ther. 2025 Jun 28;33(9):4135–42. doi: 10.1016/j.ymthe.2025.06.042 (PMC12432851; doi:10.1016/j.ymthe.2025.06.042)
Supplement: Supplementary file 1 [file mmc1.pdf]

YMTHE, Volume *33*

Supplemental Information

CD19xCD3 T cell engager blinatumomab effective in refractory generalized myasthenic syndromes

Tobias Ruck, Niklas Huntemann, Menekse Öztürk, Stefanie Schreiber, Stefanie Lichtenberg, Lars Masanneck, Christopher Nelke, Hend Ben Moussa, Thomas Ulrych, Marc Seifert, Dimitrios Mougiakakos, Sascha Dietrich, and Sven G. Meuth

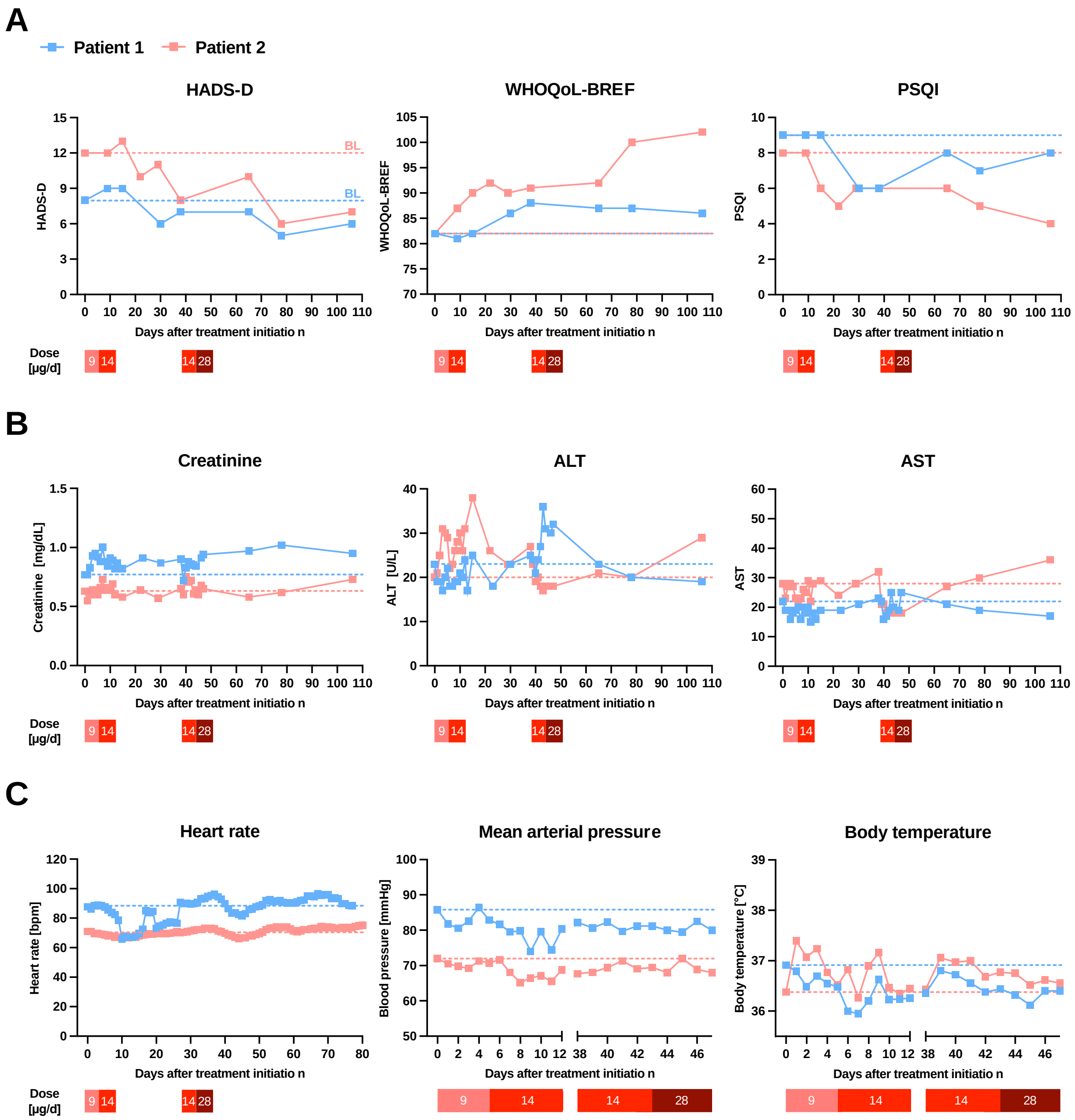

Figure S1**: Additional Efficacy and Safety Parameters on the Use of Blinatumomab in Myasthenia Gravis.** In **(A)**, patient-reported outcomes for anxiety and depression (German Hospital Anxiety and Depression Scale [HADS-D]), quality of life (World Health Organization Quality of Life Questionnaire [WHOQoL-BREF]), and sleep disturbances (Pittsburgh Sleep Quality Index [PSQI]) are depicted. For the HADS-D and PSQI, lower scores indicate better outcomes, whereas for the WHOQoL-BREF, higher scores reflect a higher quality of life. **(B)** displays creatinine, alanine aminotransferase (ALT), and aspartate aminotransferase (AST) levels over time. In **(C)**, the results of vital parameter monitoring are presented. The dotted lines indicate the respective baseline (BL) values for patient #1 (blue) and patient #2 (pink).

Videos S1-4: **Walking Tests before and after Blinatumomab Treatment.** The videos display walking tests for patient #1 **(1, 2)** and patient #2 **(3, 4)**. Videos **(1)** and **(3)** show baseline walking tests conducted shortly before the initiation of therapy. Walking tests performed 65 days after the first administration of blinatumomab are presented in videos **(2)** and **(4)**.
