# Supplementary material for: CD19xCD3 T cell engager blinatumomab effective in refractory generalized myasthenic syndromes
Source: Mol Ther. 2025 Jun 28;33(9):4135–42. doi: 10.1016/j.ymthe.2025.06.042 (PMC12432851; doi:10.1016/j.ymthe.2025.06.042)
Supplement: Supplementary file 2 [file mmc6.pdf]

**Molecular Therapy**
Case report

# CD19xCD3 T cell engager blinatumomab effective in refractory generalized myasthenic syndromes

Tobias Ruck,[1,2,3,7] Niklas Huntemann,[3,7] Menekse Öztürk,[1,2,3] Stefanie Schreiber,[3,4] Stefanie Lichtenberg,[3] Lars Masanneck,[3] Christopher Nelke,[1,2,3] Hend Ben Moussa,[3] Thomas Ulrych,[5] Marc Seifert,[5] Dimitrios Mougiakakos,[6] Sascha Dietrich,[5,7] and Sven G. Meuth[3,7]

[1]Ruhr University Bochum, BG University Hospital Bergmannsheil, Department of Neurology, 44789 Bochum, Germany; [2]BG University Hospital Bergmannsheil, Heimer Institute for Muscle Research, 44789 Bochum, Germany; [3]Department of Neurology, University Hospital and Medical Faculty Düsseldorf, Heinrich Heine University Düsseldorf, 40225 Düsseldorf, Germany; [4]Department of Neurology, Otto von Guericke University Magdeburg, 39120 Magdeburg, Germany; [5]Department of Haematology and Oncology, University Hospital Düsseldorf, and Center for Integrated Oncology Aachen Bonn Cologne, 40225 Düsseldorf, Germany; [6]Department of Haematology, Oncology, and Cell Therapy, Otto von Guericke University Magdeburg, 39120 Magdeburg, Germany

**In this case series, we report the first off-label use of the CD19xCD3 T cell engager blinatumomab in two patients with generalized myasthenia gravis (MG). Refractory MG remains a major therapeutic challenge, with patients experiencing severe disability and potentially life-threatening crises despite intensive immunotherapy. This study evaluates the clinical efficacy and safety of short-term blinatumomab treatment in two patients with severe, refractory generalized MG. Both individuals had been experiencing persistent disease burden with myasthenic crises leading to severe disability, despite multimodal immunotherapy. Following treatment with blinatumomab, both patients showed rapid and sustained clinical improvements, reflected in significant reductions in MG-specific scores (MG Activities of Daily Living scale, Quantitative MG score, and revised MG Quality of Life-15), further patient-reported outcomes, digital activity markers, and gait analyses. Laboratory findings revealed persistent B cell depletion in patient 1, whereas patient 2 demonstrated clinical improvement and autoantibody reduction despite B cell repopulation by day 106. Both patients experienced grade 1 cytokine release syndrome during initial treatment phases, but no neurotoxicity or severe adverse events were observed. This report underscores the potential of CD19xCD3 T cell engagers as a promising therapeutic approach in severe autoimmune neuroimmunological disorders, warranting further investigation in clinical trials and mechanistic studies.**

## INTRODUCTION

Myasthenia gravis (MG) is an autoantibody-mediated disorder of the neuromuscular junction (NMJ), causing fatigable muscle weakness.[1] Despite innovations in therapeutic options such as terminal complement inhibition[2–4] and antagonism of the neonatal Fc receptor,[5–7] a relevant proportion of patients still experiences a refractory disease course with potentially fatal myasthenic crises and exacerbations.[8] Recent reports have highlighted the potential of Cluster of Differentiation (CD)19-targeted chimeric antigen receptor (CAR)-T cells in patients with severe treatment-refractory MG.[9–11] However, limitations of CAR-T cell therapy, including considerable side effects, the requirement for preconditioning chemotherapy, high costs, complex logistics, and prolonged production time,[12,13] have fueled growing interest in bispecific antibodies as a promising alternative for the treatment of refractory autoimmune disorders.[14–16] As such, the CD19xCD3 bi-specific T cell engager blinatumomab, originally developed for the therapy of acute lymphoblastic leukemia, consists of two single-chain variable fragments that specifically target CD3 (C-terminal) and CD19 (N-terminal).[17,18] By bridging T and B cells, blinatumomab facilitates T cell engagement and the release of perforins, caspases, and granzymes, inducing B cell apoptosis.[19,20] Additionally, blinatumomab achieves a higher degree of tissue penetration and induces deeper B cell depletion compared to standard B cell depleting agents such as rituximab.[14,21] Here, we present the first report on the use of blinatumomab for the treatment of therapy-refractory MG.

### Patient details and clinical findings

The 41-year-old female patient 1 was diagnosed with MG in August 2020. Extensive laboratory diagnostics failed to detect myasthenia-associated autoantibodies. Given the typical clinical presentation, an intercostal muscle biopsy was performed to support the diagnosis,[22] revealing characteristic MG pathology with immunoglobulin G1 (IgG1) and complement deposits at the NMJ. Of note, autoantibody testing was repeated before blinatumomab treatment and remained negative. The disease initially manifested with fluctuating ptosis and diplopia, followed shortly by generalized muscle weakness, dysphagia, and exertional dyspnea. Neurological examination revealed positive Simpson and ice-pack tests, whereas repetitive nerve stimulation showed no pathological decrement. The patient

Received 18 February 2025; accepted 26 June 2025;
https://doi.org/10.1016/j.ymthe.2025.06.042.

[7]These authors contributed equally

**Correspondence:** Tobias Ruck, Ruhr University Bochum, BG University Hospital Bergmannsheil, Department of Neurology, 44789 Bochum, Germany.
**E-mail:** tobias.ruck@med.uni-duesseldorf.de


Published by Elsevier Inc. on behalf of The American Society of Gene and Cell Therapy.
This is an open access article under the CC BY license (http://creativecommons.org/licenses/by/4.0/).

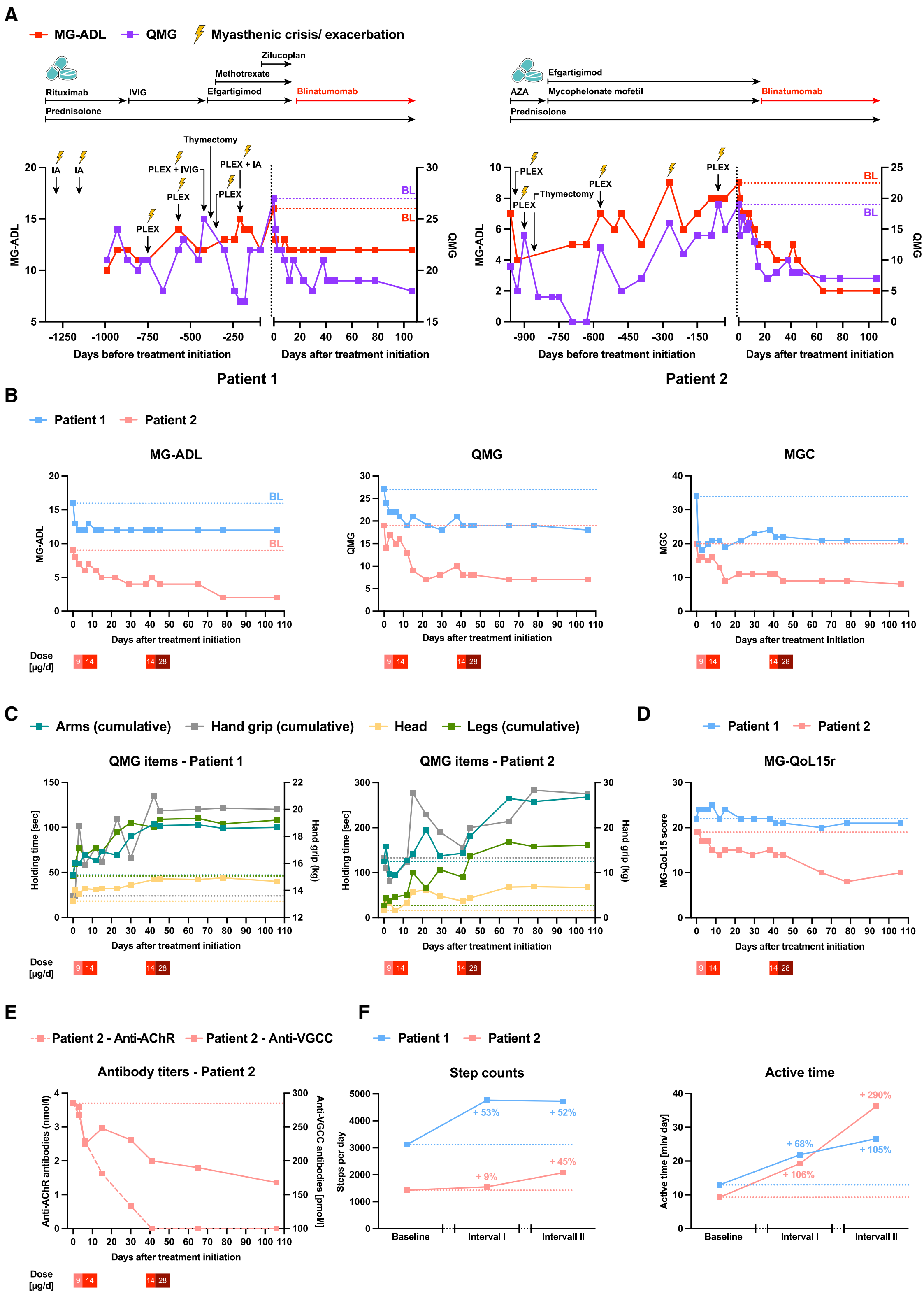


(legend on next page)

exhibited marked clinical improvement following symptomatic treatment with pyridostigmine. An extensive diagnostic evaluation, including imaging and serological screening, found no alternative etiology. The relevant comorbidities of patient 1 included Raynaud's phenomenon, erythema nodosum, arthritis, and interface dermatitis. Despite a multifaceted therapeutic regimen (pyridostigmine, rituximab [3 cycles, relative CD19 lymphocyte proportion of <1%], prednisolone [dose range: 5–60 mg/day], immunoadsorption, intravenous immunoglobulins, plasma exchange [PLEX], methotrexate [MTX], efgartigimod, zilucoplan, and thymectomy), this patient experienced a severe disease course, with myasthenic exacerbations and crises culminating in profound disability (Figure 1A; Table 1). Due to the high symptom burden with spontaneous diplopia, ptosis, significant reductions in vital capacity to 42% of the predicted value, and a tetraparesis resulting in a maximal Myasthenia Gravis Foundation of America (MGFA) class of IVa, we decided to treat the patient with blinatumomab.

Patient 2, a 60-year-old woman, was diagnosed in October 2021 with an anti-acetylcholine receptor (AChR) antibody-positive MG and comorbid Lambert-Eaton myasthenic syndrome, confirmed by the presence of anti-voltage-gated calcium channel (VGCC) P/Q-type antibodies. The initial titers at diagnosis measured 20.8 nmol/L for anti-AChR and 354.7 pmol/L for anti-VGCC antibodies, as determined by enzyme-linked immunosorbent assay and radioimmunoassay, respectively. At disease onset she presented with proximally predominant tetraparesis that worsened with exercise and over the course of the day, together with dysphagia for solid food, exertional dyspnea, fatigability during prolonged speech, and bilateral ptosis. Neurological examination additionally demonstrated pronounced post-exercise reflex facilitation, and repetitive nerve stimulation revealed both a pathological decrement and increment. Patient 2 experienced recurrent myasthenic exacerbations as well as crises under intensive symptomatic treatment and immunotherapy with prednisolone (dose range: 5–60 mg/day), pyridostigmine, amifampridine, azathioprine, mycophenolate mofetil, PLEX, efgartigimod, and thymectomy, reaching a maximal MGFA class of IVb (Figure 1A; Table 1). Essentially, patient 2 had not been exposed to any prior B cell-depleting therapy. Given the persistent, functionally relevant symptoms characterized by proximally pronounced tetraparesis, severe mobility impairment, and a myasthenic crisis in August 2024, treatment with blinatumomab was initiated.

Ten days prior to blinatumomab therapy, concurrent MG medication was discontinued, except for prednisolone and symptomatic treatment. After obtaining informed written consent, each patient received two cycles of blinatumomab at 26-day intervals. Blinatumomab was administered as a continuous intravenous infusion for 12 consecutive days per cycle. The first cycle comprised a dosing regimen of 9 μg/day on days 1–5, followed by 14 μg/day on days 6–12, resulting in a cumulative dose of 143 μg. In the second cycle, the daily dose was increased to 14 μg/day on days 1–5 and 28 μg/day on days 6–12, yielding a cumulative dose of 266 μg. To prevent cytokine release syndrome (CRS), patients underwent prophylactic treatment with 20 mg dexamethasone and 1 g paracetamol, administered 1 h prior to the start of each treatment cycle.

Clinical assessment comprised MG-specific disease scores including the Quantitative MG (QMG), MG Activities of Daily Living (MG-ADL), MG Composite (MGC), and the 15-item revised MG Quality of Life questionnaire (MG-QoL15r). Additionally, assessments of the Pittsburgh Sleep Quality Index, World Health Organization Quality of Life Questionnaire, and the German version of the Hospital Anxiety and Depression Scale were conducted. Patients were continuously monitored using wearables. Wearable data for patient 1 were extracted from the patient's privately owned Apple Watch Series 7 at their request via the HealthKit Application Programming Interface (API), capturing heart rate, step count, and activity metrics. Data for patient 2 were obtained from a Withings Smartwatch 2 provided as part of an observational study at University Hospital Düsseldorf, using the Withings API under standard OAuth2 authentication to retrieve the same set of physiological parameters. In addition, standard laboratory parameters, including autoantibody levels, were evaluated closely.

Both patients demonstrated rapid and sustained improvements in MG-ADL and QMG scores following treatment with blinatumomab (Figures 1A and 1B). By the end of the first treatment cycle, MG-ADL scores decreased from 16 to 12 in patient 1 and from 9 to 6 in patient 2. During the follow-up period, the MG-ADL remained stable in patient 1, while patient 2 showed further improvements, reaching a score of 2. In addition to enhancements in ADLs, clinical examinations demonstrated significant regression of myasthenic symptoms. In patient 1, the QMG score declined from 27 to 18 points after 30 days, with 6 points attributed to stable ocular symptoms, and continued to show sustained improvement over the following

**Figure 1. Efficacy of blinatumomab in myasthenia gravis**

(A) The medical history of patient 1 (left) and patient 2 (right) is presented, beginning at the time of diagnosis. Shown are the immunomodulatory therapies administered, interventions for myasthenic exacerbations or crises, the timing of thymectomy, and the Myasthenia Gravis Activities of Daily Living (MG-ADL) as well as Quantitative MG (QMG) scores before and after the initiation of blinatumomab treatment. (B) MG-ADL and QMG in absolute values and Myasthenia Gravis Composite (MGC) scores, reflecting disease severity and therapeutic effects. (C) The development of arm-, leg-, and head-lifting times, as well as hand grip strength, as assessed during QMG evaluation. In contrast to the MG-ADL, QMG, revised 15-item Myasthenia Gravis Quality of Life questionnaire (MG-QoL15r), and MGC, where lower scores indicate clinical improvement and higher values for the depicted QMG items reflect functional recovery. (D) Longitudinal changes in the MG-QoL15r. (E) Course of anti-acetylcholine receptor (AChR) and anti-voltage-gated calcium channels (VGCC) antibody levels in patient 2 over time. Due to the seronegative status of patient 1, autoantibody levels are shown only for patient 2. (F) Real-world activity as well as daily step counts, as recorded by wearable devices. "Baseline" (BL) is defined as the time period prior to initiation of blinatumomab therapy. "Interval I" denotes the period between the first and second cycle, and "Interval II" denotes the period after the second cycle. The mean values for each interval are presented. The dotted line indicates the BL values for (A) MG-ADL (red) and QMG (violet) and, in (B–F), for patient 1 (blue) and patient 2 (pink).

**Table 1. Baseline characteristics of patients 1 and 2**

| Baseline patient characteristics | Patient 1 | Patient 2 |
|---|---|---|
| Sex | female | female |
| Age at baseline, y | 41 | 59 |
| Age at diagnosis, y | 37 | 57 |
| Disease duration, months | 51 | 36 |
| Autoantibody profile | | |
| Acetylcholine receptor (live cell-based assay) | – | + |
| Low-density lipoprotein receptor-related protein 4 | – | – |
| Muscle-specific tyrosine kinase | – | – |
| Ryanodine receptor | – | not tested |
| SOX1 | not tested | – |
| Titin | – | – |
| Voltage-gated calcium channel P/Q type | – | + |
| Neurophysiological examination | | |
| Decrement | – | + |
| Increment | – | + |
| Muscle biopsy | immunoglobulin G1 and complement deposits at the neuromuscular junction | not tested |
| Thymectomy | + | + |
| Thymoma | – | – |
| Response to oral pyridostigmine | + | + |
| Comorbidities | arthritis, hypothyroidism, interface dermatitis, paroxysmal atrial fibrillation, Raynaud's phenomenon | adjustment disorder, gastritis, osteoporosis |
| Previous therapies (disease duration, months) | | |
| Amifampridine | – | + (34) |
| Azathioprine | – | + (5) |
| Efgartigimod | + (6) | + (28) |
| Immunoadsorption | + | – |
| Intravenous immunoglobulins | + (13) | – |
| Methotrexate | + (14) | – |
| Mycophenolate mofetil | – | + (27) |
| Plasmapheresis | + | + |
| Prednisolon (dose range, minimum–maximum) | + (50) (5–60 mg) | + (34) (5–60 mg/day) |
| Pyridostigmine | + (50) | + (32) |
| Rituximab | + (13) | – |
| Zilucoplan | + (5) | – |

Baseline is defined as the time of blinatumomab initiation. + indicates a positive finding or prior therapy with the respective drug, whereas – denotes a negative test or absence of prior therapy. The number in parentheses represents the duration of therapy in months.

3 months (Figures 1A and 1B). Analysis of individual QMG items confirmed a marked treatment response with a 2-fold increase in arm-, leg-, and head-lifting times, as well as in grip strength (Figure 1C). Patient 2 experienced more pronounced improvements, with a QMG score reduction from 19 to 8 within the first month, followed by sustained stabilization at 7 points after 3 months (Figures 1A and 1B). Residual paresis of axial muscle groups accounted for the majority of the remaining QMG score items. At the 3-month follow-up, patient 2 required no immunotherapy other than corticosteroids, while patient 1 received additional MTX. Moreover, clinical amelioration correlated with further patient-reported outcomes in both patients (Figures 1D and S1A) and autoantibody titers in patient 2 (Figure 1E). Disease improvement was also reflected in a significant enhancement in gait function (Videos S1, S2, S3, and S4), as well as an increase in step counts and activity markers in the continuous digital monitoring (Figure 1F). Of note,

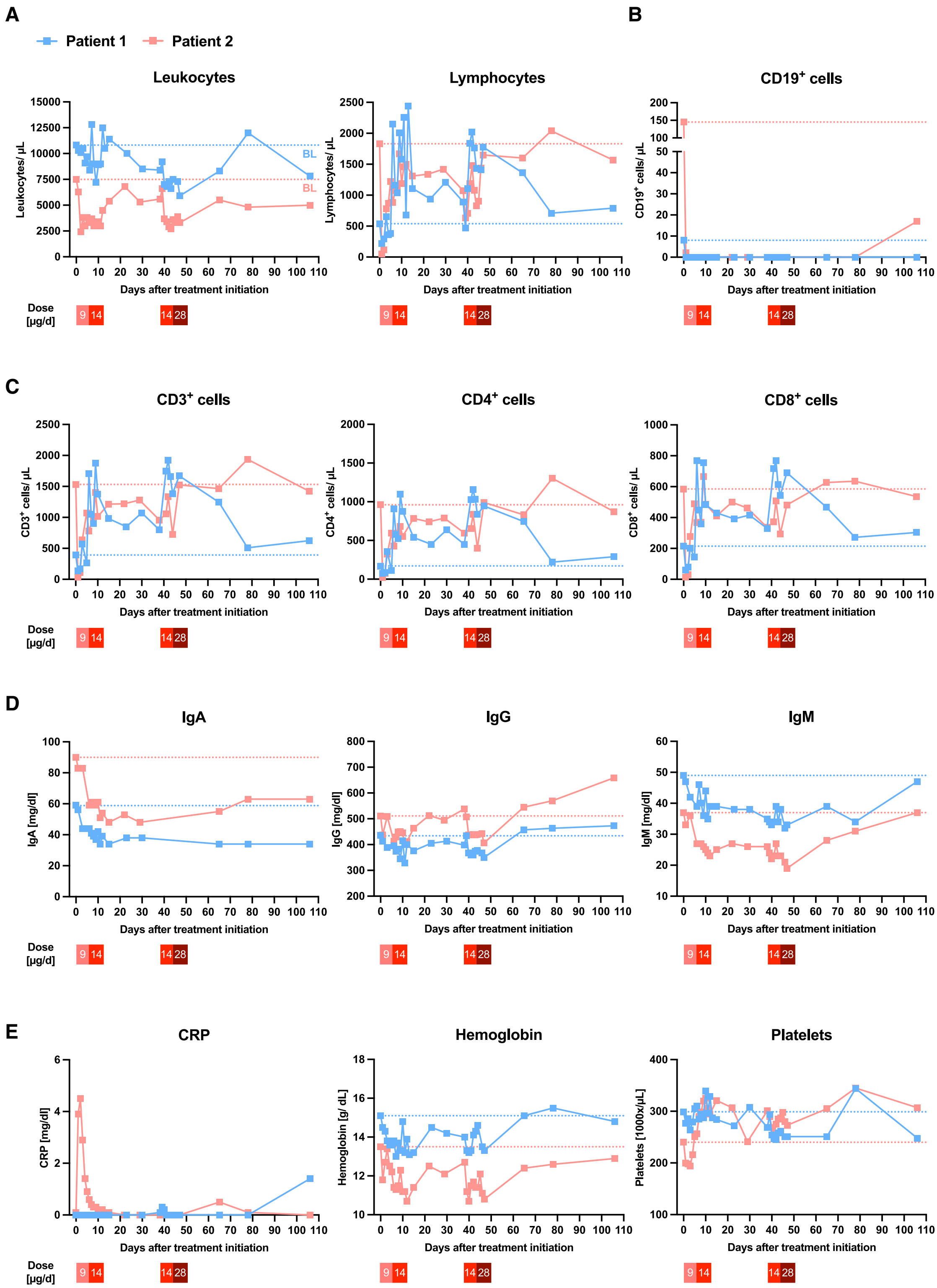


(legend on next page)

patient 1 exhibited a marked regression of additional autoimmune phenomena, evidenced by a remission of erythema nodosum and a substantial amelioration of arthralgias.

Laboratory results revealed a rapid decrease in leukocyte and lymphocyte counts with persistent B cell depletion in patient 1 (Figures 2A and 2B). In patient 2, clinical improvements and reduction of autoantibody levels continued despite B cell repopulation on day 106. Effects on Ig levels remained minimal (Figures 2B and 2D). The initial reduction in T cells may indicate migration from the blood into the tissue (Figure 2C). Further laboratory investigations and measurements of vital parameters remained without relevant findings (Figures 2E, S1B, and S1C).

In terms of safety, both patients experienced grade 1 CRS, characterized by mild fever (Figure S1C), general malaise, and headaches during the initial days of each cycle and dose escalation. Patient 1 reported transient diarrhea and worsening of preexisting tachycardia during the first cycle. There were no signs of immune effector cell-associated neurotoxicity syndrome (ICANS).

## DISCUSSION

This report underscores the potential of short-term treatment with CD19-targeting T cell engagers in autoimmune neurological disorders such as refractory MG. Both previously treatment-resistant patients exhibited a rapid improvement in MG-specific scores, including MG-ADL and QMG. Blinatumomab therapy was generally well tolerated, with only mild CRS reported and no evidence of ICANS.

Notably, in patient 2, the latest follow-up showed B cell reappearance along with a continued increase in IgG levels above baseline, likely due to prior treatment with efgartigimod and recent plasmapheresis. Interestingly, this was accompanied by a further drop in autoantibody levels, which may suggest a beneficial repopulation of B cells with ongoing depletion of autoreactive clones. Further mechanistic studies are required to validate this hypothesis. Additionally, some questions remain regarding the use of blinatumomab in refractory MG, including the optimal number of treatment cycles and the appropriate duration of therapy-free intervals. Moreover, given that autoantibodies may also play a pivotal role in muscle-specific kinase and low-density lipoprotein receptor-related protein 4-associated myasthenia, blinatumomab could demonstrate clinical efficacy in these subtypes as well.[1]

Since this first-in-human experience comprises two cases, our findings should be viewed as proof-of-concept rather than evidence of broad clinical efficacy. The small sample size precludes statistical analysis. This is compounded by the all-female composition of the cohort and 3-month follow-up, which prevents assessment of long-term safety aspects. Moreover, the two patients differed in autoantibody status, prior immunotherapies, and comorbidities, introducing clinical heterogeneity. To confirm the encouraging signals of this study, prospective randomized trials enrolling patients with refractory MG despite treatment with approved high-efficacy agents are needed. Such studies should also aim to optimize blinatumomab dosing and cycle intervals. In parallel, mechanistic investigations, incorporating deep immune phenotyping, flow cytometric analyses, and sequencing of T and B cell clonal dynamics, are essential to elucidate patterns of immune repopulation and, ideally, to identify predictive biomarkers of clinical response.

In contrast to classical CD20-directed agents such as rituximab, blinatumomab eliminates B cells via a mechanistically distinct pathway. While rituximab depends mainly on complement- and Fc receptor-mediated cytotoxicity, blinatumomab redirects T cells to CD19-expressing targets, including antibody-producing plasmablasts and long-lived plasma that escape CD20 depletion, and might therefore permit a more profound depletion of the B cell compartment.[19,20,23–25]

Compared to CAR-T cell therapy, blinatumomab provides several advantages. These may include an improved controllability due to its short half-life of approximately 2 h.[12,13,26] Additionally, blinatumomab is associated with lower treatment costs and does not require preconditioning chemotherapy, making it a more accessible therapeutic option. However, the limited number of reported cases precludes drawing reliable conclusions about their comparative efficacy.

This report warrants further evaluation to assess the long-term efficacy, safety profiles, and mechanistic pathways of blinatumomab in MG in controlled clinical trials.

## DATA AVAILABILITY

All data will be made available to researchers and clinicians upon reasonable request to the lead contact.

## ACKNOWLEDGMENTS

The conduct of the study and/or the preparation of the manuscript was funded by the German Research Foundation (DFG; to T.R.: RU 2169/5-1 and 417677437/GRK2578; to C.N.: NE 2774/2-1) and by the EFRE/JTF program (B2B-RARE; to T.R.: EFRE-20800340). None of the funding sources was involved in any way in the study design; the collection, analysis, and interpretation of data; the writing of the report; or the decision to submit the paper for publication.

## AUTHOR CONTRIBUTIONS

T.R., N.H., M.Ö., S.D., and S.G.M. designed the study. Clinical data were provided by T. R., N.H., M.Ö., L.M., C.N., H.B.M., and S.G.M. Formal analyses were done by T.R. and N.H. Figures were created by N.H. Data were interpreted by T.R., N.H., M.Ö., S.S., S.L., L.M., C.N., H.B.M., T.U., M.S., D.M., S.D., and S.G.M. Resources were provided by T.R.,

**Figure 2. Safety of blinatumomab in MG**

(A) Leukocytes and lymphocyte levels. (B) Changes in Cluster of Differentiation (CD)19$^{+}$ B cell counts are depicted. (C) CD3$^{+}$, CD4$^{+}$, and CD8$^{+}$ T cell counts during blinatumomab treatment. (D) Changes in immunoglobulin (Ig)A, IgG, and IgM concentrations in the blood. (E) C-reactive protein (CRP), hemoglobin levels, and platelet counts over the course of therapy. Baseline (BL) values for patient 1 (blue) and patient 2 (pink) are indicated by dotted lines.

S.D., and S.G.M. T.R. and N.H. wrote the original draft. M.Ö., S.S., S.L., L.M., C.N., H.B. M., T.U., M.S., D.M., S.D., and S.G.M. critically reviewed and edited the manuscript. S.D. and S.G.M. supervised the study. The guarantors of the study are T.R. and S.G.M.

## DECLARATION OF INTERESTS

T.R. received honoraria for lecturing, consulting, travel expenses for attending meetings, and research support from Alexion, Argenx, Biogen, BMS, Celgene, Genzyme, J&J, Merck, Novartis, Roche, Sanofi, and UCB. His research was funded by the Deutsche Gesellschaft für Muskelkranke (DGM) e.V., German Ministry for Education and Research (BMBF), German Research Foundation (DFG), Else Kroner-Fresenius Foundation, and Interdisciplinary Center for Clinical Studies (IZKF) Muenster, all outside the scope of this work. N.H. received honoraria for lecturing, consulting, travel expenses for attending meetings, and research support from Alexion, ArgenX, Janssen-Cilag, Merck, Novartis, UCB, and Viatris. His research has been funded by the DFG, outside the scope of this work. M.Ö. received honoraria for lecturing, travel expenses for attending meetings, and research support from Amicus Therapeutics, Argenx, CSL Behring, and Novartis. S.S. received research support from the DFG, Else Kroner-Fresenius Foundation, and the Federal Ministry for Economic Affairs and Energy. L.M. received honoraria for lecturing, consulting, travel expenses for attending meetings, and research support from Alexion, Argenx, B. Braun Foundation, Biogen, Merck, Neuraxpharm, Novartis, Roche, and Sanofi. His research was funded by the DFG and Deutsch Multiple Sklerose Gesellschaft (German Multiple Sclerosis Foundation), all outside the scope of this work. He is chair of the German Society for Digital Medicine. C.N. received honoraria for lecturing and consulting from Alexion and ArgenX. D.M. received honoraria for lecturing, consulting, and travel expenses for attending meetings from AbbVie, AstraZeneca, AvenCell, Beigne, BMS, Galapagos, Gilead, Janssen, Lilly, Kyverna Therapeutics, Miltenyi Novartis, Pfizer, and Roche. S.D. received honoraria for lecturing, consulting, travel expenses for attending meetings, and research support from BeiGene, BMS, Celgene, Johnson & Johnson, Kilte/ Gliead, Novartis, Pierre Fabre, and Roche. His research was supported by the BMBF (German Ministry of Education and Research) and DFG. S.G.M. received honoraria for lecturing, consulting, travel expenses for attending meetings, and research support from Academy 2, Argenx, Alexion, Almirall, Amicus Therapeutics Germany, AstraZeneca, Bayer HealthCare, Biogen, BioNTech, BMS, Celgene, Datamed, Demecan, Desitin, Diamed, Diaplan, DIU Dresden, DPmed, Gen Medicine and Healthcare products, Genzyme, Hexal AG, IGES, Impulze GmbH, Janssen Cilag, KW Medipoint, MedDay Pharmaceuticals, Medmile, Merck Serono, MICE, Mylan, Neuraxpharm, Neuropoint, Novartis, Novo Nordisk, ONO Pharma, Oxford PharmaGenesis, QuintilesIMS, Roche, Sanofi, Springer Medizin Verlag, STADA, Chugai Pharma, Teva, UCB, Viatris, Wings for Life International, and Xcenda. His research is funded by the BMBF, German Federal Institute for Risk Assessment, DFG, Else Kroner-Fresenius Foundation, Gemeinsamer Bundesausschuss, German Academic Exchange Service, Hertie Foundation, IZKF Muenster, German Foundation for Neurology, Ministry of Culture and Science of the State of North Rhine-Westphalia, the Daimler and Benz Foundation, DMSG (German Society for Multiple Sclerosis), Peek & Cloppenburg Düsseldorf Foundation, Hempel Foundation for Science, Art and Welfare, German Alzheimer Society e.V. and Alexion, Almirall, Amicus Therapeutics Germany, Argenx, Bayer Vital GmbH, BGP Products Operations (Viatris Company), Biogen, BMS, Demecan, Diamed, DGM e.v., Fresenius Medical Care, Genzyme, Gesellschaft von Freunden und Förderern der Heinrich-Heine-Universität Düsseldorf e.V., HERZ Burgdorf, Hexal, Janssen, Merck Serono, Novartis, Novo Nordisk Pharma, ONO Pharma, Roche, and Teva.

## SUPPLEMENTAL INFORMATION

Supplemental information can be found online at https://doi.org/10.1016/j.ymthe.2025.06.042.

## REFERENCES


1. Gilhus, N.E., Tzartos, S., Evoli, A., Palace, J., Burns, T.M., and Verschuuren, J.J.G.M. (2019). Myasthenia gravis. Nat. Rev. Dis. Primers *5*, 30. https://doi.org/10.1038/s41572-019-0079-y.
2. Howard, J.F., Utsugisawa, K., Benatar, M., Murai, H., Barohn, R.J., Illa, I., Jacob, S., Vissing, J., Burns, T.M., Kissel, J.T., et al. (2017). Safety and efficacy of eculizumab in anti-acetylcholine receptor antibody-positive refractory generalised myasthenia gravis (REGAIN): a phase 3, randomised, double-blind, placebo-controlled, multicentre study. Lancet Neurol. *16*, 976–986. https://doi.org/10.1016/S1474-4422(17)30369-1.
3. Vu, T., Meisel, A., Mantegazza, R., Annane, D., Katsuno, M., Aguzzi, R., Enayetallah, A., Beasley, K.N., Rampal, N., and Howard, J.F. (2022). Terminal Complement Inhibitor Ravulizumab in Generalized Myasthenia Gravis. NEJM Evid. *1*, EVIDoa2100066. https://doi.org/10.1056/EVIDoa2100066.
4. Howard, J.F., Bresch, S., Genge, A., Hewamadduma, C., Hinton, J., Hussain, Y., Juntas-Morales, R., Kaminski, H.J., Maniaol, A., Mantegazza, R., et al. (2023). Safety and efficacy of zilucoplan in patients with generalised myasthenia gravis (RAISE): a randomised, double-blind, placebo-controlled, phase 3 study. Lancet Neurol. *22*, 395–406. https://doi.org/10.1016/S1474-4422(23)00080-7.
5. Howard, J.F., Bril, V., Vu, T., Karam, C., Peric, S., Margania, T., Murai, H., Bilinska, M., Shakarishvili, R., Smilowski, M., et al. (2021). Safety, efficacy, and tolerability of efgartigimod in patients with generalised myasthenia gravis (ADAPT): a multicentre, randomised, placebo-controlled, phase 3 trial. Lancet Neurol. *20*, 526–536. https://doi.org/10.1016/S1474-4422(21)00159-9.
6. Bril, V., Drużdż, A., Grosskreutz, J., Habib, A.A., Mantegazza, R., Sacconi, S., Utsugisawa, K., Vissing, J., Vu, T., Boehnlein, M., et al. (2023). Safety and efficacy of rozanolixizumab in patients with generalised myasthenia gravis (MycarinG): a randomised, double-blind, placebo-controlled, adaptive phase 3 study. Lancet Neurol. *22*, 383–394. https://doi.org/10.1016/S1474-4422(23)00077-7.
7. Yan, C., Yue, Y., Guan, Y., Bu, B., Ke, Q., Duan, R., Deng, H., Xue, Q., Jiang, H., Xiao, F., et al. (2024). Batoclimab vs Placebo for Generalized Myasthenia Gravis: A Randomized Clinical Trial. JAMA Neurol. *81*, 336–345. https://doi.org/10.1001/jamaneurol.2024.0044.
8. Huntemann, N., Gerischer, L., Herdick, M., Nelke, C., Stascheit, F., Hoffmann, S., Öztürk, M., Schroeter, C.B., Lehnerer, S., Stein, M., et al. (2025). C5 complement inhibition versus FcRn modulation in generalised myasthenia gravis. J. Neurol. Neurosurg. Psychiatry *96*, 310–321. https://doi.org/10.1136/jnnp-2024-334404.
9. Granit, V., Benatar, M., Kurtoglu, M., Miljković, M.D., Chahin, N., Sahagian, G., Feinberg, M.H., Slansky, A., Vu, T., Jewell, C.M., et al. (2023). Safety and clinical activity of autologous RNA chimeric antigen receptor T-cell therapy in myasthenia gravis (MG-001): a prospective, multicentre, open-label, non-randomised phase 1b/2a study. Lancet Neurol. *22*, 578–590. https://doi.org/10.1016/S1474-4422(23)00194-1.
10. Haghikia, A., Hegelmaier, T., Wolleschak, D., Böttcher, M., Desel, C., Borie, D., Motte, J., Schett, G., Schroers, R., Gold, R., and Mougiakakos, D. (2023). Anti-CD19 CAR T cells for refractory myasthenia gravis. Lancet Neurol. *22*, 1104–1105. https://doi.org/10.1016/S1474-4422(23)00375-7.
11. Motte, J., Sgodzai, M., Schneider-Gold, C., Steckel, N., Mika, T., Hegelmaier, T., Borie, D., Haghikia, A., Mougiakakos, D., Schroers, R., and Gold, R. (2024). Treatment of concomitant myasthenia gravis and Lambert-Eaton myasthenic syndrome with autologous CD19-targeted CAR T cells. Neuron *112*, 1757–1763.e2. https://doi.org/10.1016/j.neuron.2024.04.014.
12. Kobbe, G., Brüggemann, M., Baermann, B.N., Wiegand, L., Trautmann, H., Yousefian, S., Libertini, S., Menssen, H.D., Maier, H.J., Ulrich, P., et al. (2024). Aggressive Lymphoma after CD19 CAR T-Cell Therapy. N. Engl. J. Med. *391*, 1217–1226. https://doi.org/10.1056/NEJMoa2402730.
13. Ismail, F.S., Gallus, M., Meuth, S.G., Okada, H., Hartung, H.P., and Melzer, N. (2025). Current and Future Roles of Chimeric Antigen Receptor T-Cell Therapy in Neurology: A Review. JAMA Neurol. *82*, 93–103. https://doi.org/10.1001/jamaneurol.2024.3818.
14. Bucci, L., Hagen, M., Rothe, T., Raimondo, M.G., Fagni, F., Tur, C., Wirsching, A., Wacker, J., Wilhelm, A., Auger, J.P., et al. (2024). Bispecific T cell engager therapy for refractory rheumatoid arthritis. Nat. Med. *30*, 1593–1601. https://doi.org/10.1038/s41591-024-02964-1.
15. Hagen, M., Bucci, L., Böltz, S., Nöthling, D.M., Rothe, T., Anoshkin, K., Raimondo, M.G., Tur, C., Wirsching, A., Wacker, J., et al. (2024). BCMA-Targeted T-Cell-Engager Therapy for Autoimmune Disease. N. Engl. J. Med. *391*, 867–869. https://doi.org/10.1056/NEJMc2408786.
16. Alexander, T., Krönke, J., Cheng, Q., Keller, U., and Krönke, G. (2024). Teclistamab-Induced Remission in Refractory Systemic Lupus Erythematosus. N. Engl. J. Med. *391*, 864–866. https://doi.org/10.1056/NEJMc2407150.
17. Kantarjian, H., Stein, A., Gökbuget, N., Fielding, A.K., Schuh, A.C., Ribera, J.M., Wei, A., Dombret, H., Foà, R., Bassan, R., et al. (2017). Blinatumomab versus

Chemotherapy for Advanced Acute Lymphoblastic Leukemia. N. Engl. J. Med. *376*, 836–847. https://doi.org/10.1056/NEJMoa1609783.

18. Löffler, A., Kufer, P., Lutterbüse, R., Zettl, F., Daniel, P.T., Schwenkenbecher, J.M., Riethmüller, G., Dörken, B., and Bargou, R.C. (2000). A recombinant bispecific single-chain antibody, CD19 x CD3, induces rapid and high lymphoma-directed cytotoxicity by unstimulated T lymphocytes. Blood *95*, 2098–2103.
19. Gruen, M., Bommert, K., and Bargou, R.C. (2004). T-cell-mediated lysis of B cells induced by a CD19xCD3 bispecific single-chain antibody is perforin dependent and death receptor independent. Cancer Immunol. Immunother. *53*, 625–632. https://doi.org/10.1007/s00262-003-0496-2.
20. Hoffmann, P., Hofmeister, R., Brischwein, K., Brandl, C., Crommer, S., Bargou, R., Itin, C., Prang, N., and Baeuerle, P.A. (2005). Serial killing of tumor cells by cytotoxic T cells redirected with a CD19-/CD3-bispecific single-chain antibody construct. Int. J. Cancer *115*, 98–104. https://doi.org/10.1002/ijc.20908.
21. d'Argouges, S., Wissing, S., Brandl, C., Prang, N., Lutterbuese, R., Kozhich, A., Suzich, J., Locher, M., Kiener, P., Kufer, P., et al. (2009). Combination of rituximab with blinatumomab (MT103/MEDI-538), a T cell-engaging CD19-/CD3-bispecific antibody, for highly efficient lysis of human B lymphoma cells. Leuk. Res. *33*, 465–473. https://doi.org/10.1016/j.leukres.2008.08.025.
22. Hoffmann, S., Harms, L., Schuelke, M., Rückert, J.C., Goebel, H.H., Stenzel, W., and Meisel, A. (2020). Complement deposition at the neuromuscular junction in seronegative myasthenia gravis. Acta Neuropathol. *139*, 1119–1122. https://doi.org/10.1007/s00401-020-02147-5.
23. Lee, D.S.W., Rojas, O.L., and Gommerman, J.L. (2021). B cell depletion therapies in autoimmune disease: advances and mechanistic insights. Nat. Rev. Drug Discov. *20*, 179–199. https://doi.org/10.1038/s41573-020-00092-2.
24. Forsthuber, T.G., Cimbora, D.M., Ratchford, J.N., Katz, E., and Stüve, O. (2018). B cell-based therapies in CNS autoimmunity: differentiating CD19 and CD20 as therapeutic targets. Ther. Adv. Neurol. Disord. *11*, 1756286418761697. https://doi.org/10.1177/1756286418761697.
25. Chan, A.C., and Carter, P.J. (2010). Therapeutic antibodies for autoimmunity and inflammation. Nat. Rev. Immunol. *10*, 301–316. https://doi.org/10.1038/nri2761.
26. Klinger, M., Brandl, C., Zugmaier, G., Hijazi, Y., Bargou, R.C., Topp, M.S., Gökbuget, N., Neumann, S., Goebeler, M., Viardot, A., et al. (2012). Immunopharmacologic response of patients with B-lineage acute lymphoblastic leukemia to continuous infusion of T cell-engaging CD19/CD3-bispecific BiTE antibody blinatumomab. Blood *119*, 6226–6233. https://doi.org/10.1182/blood-2012-01-400515.

YMTHE, Volume *33*

## Supplemental Information

# CD19xCD3 T cell engager blinatumomab effective in refractory generalized myasthenic syndromes

**Tobias Ruck, Niklas Huntemann, Menekse Öztürk, Stefanie Schreiber, Stefanie Lichtenberg, Lars Masanneck, Christopher Nelke, Hend Ben Moussa, Thomas Ulrych, Marc Seifert, Dimitrios Mougiakakos, Sascha Dietrich, and Sven G. Meuth**

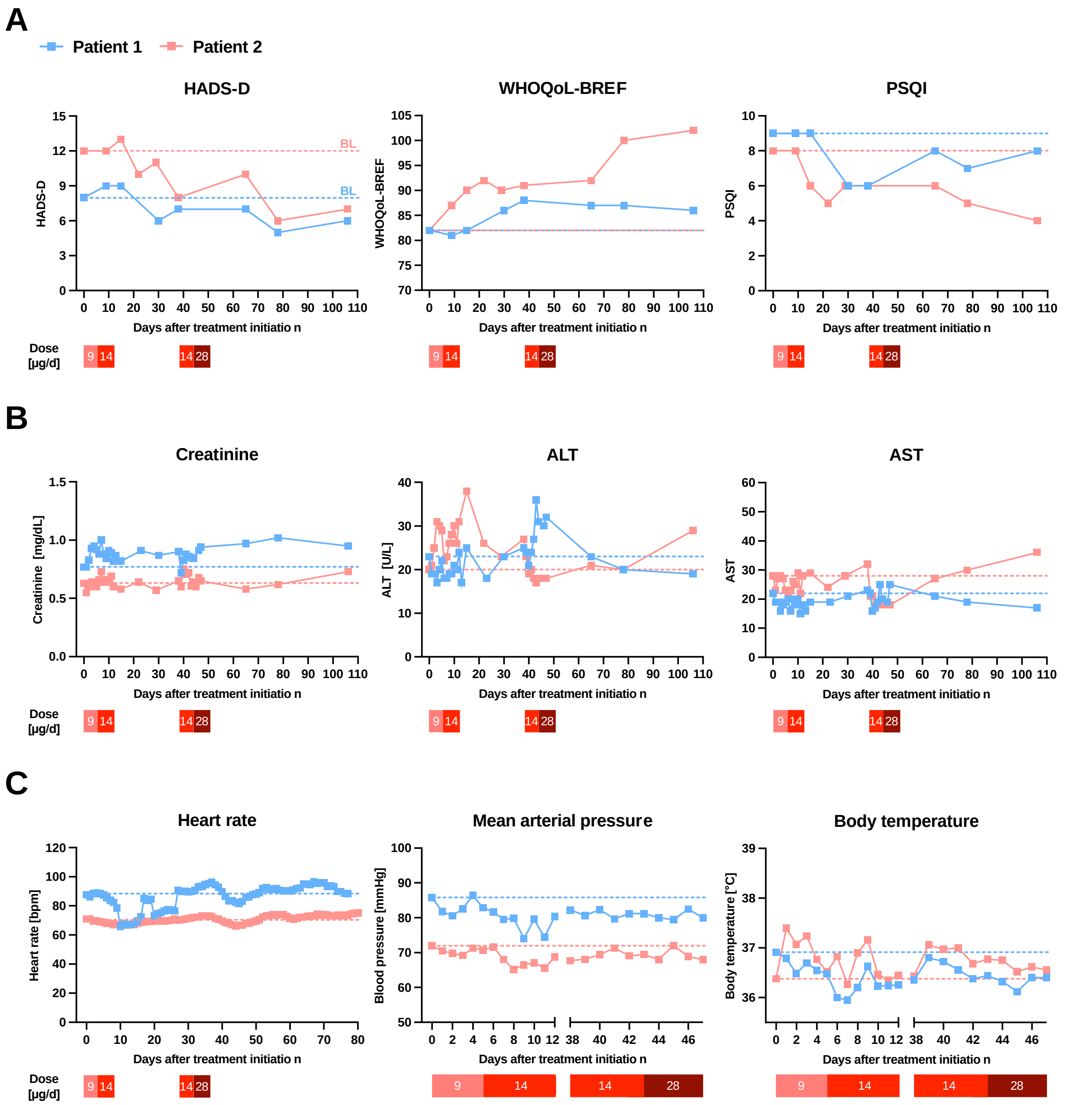


***Figure S1*: Additional Efficacy and Safety Parameters on the Use of Blinatumomab in Myasthenia Gravis.** In **(A)**, patient-reported outcomes for anxiety and depression (German Hospital Anxiety and Depression Scale [HADS-D]), quality of life (World Health Organization Quality of Life Questionnaire [WHOQoL-BREF]), and sleep disturbances (Pittsburgh Sleep Quality Index [PSQI]) are depicted. For the HADS-D and PSQI, lower scores indicate better outcomes, whereas for the WHOQoL-BREF, higher scores reflect a higher quality of life. **(B)** displays creatinine, alanine aminotransferase (ALT), and aspartate aminotransferase (AST) levels over time. In **(C)**, the results of vital parameter monitoring are presented. The dotted lines indicate the respective baseline (BL) values for patient #1 (blue) and patient #2 (pink).

***Videos S1-4:*** **Walking Tests before and after Blinatumomab Treatment.** The videos display walking tests for patient #1 **(1, 2)** and patient #2 **(3, 4)**. Videos **(1)** and **(3)** show baseline walking tests conducted shortly before the initiation of therapy. Walking tests performed 65 days after the first administration of blinatumomab are presented in videos **(2)** and **(4)**.
